# Clinical value of nedaplatin-based chemotherapy combined with radiotherapy for locoregional advanced nasopharyngeal carcinoma: a retrospective, propensity score-matched analysis

**DOI:** 10.7150/jca.47090

**Published:** 2020-09-30

**Authors:** Ze-Jiang Zhan, Hao-Yun Tao, Wen-Ze Qiu, Zhong-Yuan Liu, Rui-Xin Zhang, Kai Liao, Guo Li, Ya-Wei Yuan, Tai-Ze Yuan, Rong-Hui Zheng

**Affiliations:** 1 Department of Radiation Oncology, Affiliated Cancer Hospital & Institute of Guangzhou Medical University, Guangdong, P. R. China.; 2 Department of Radiation Oncology, Guangzhou Concord Cancer Center, Guangdong, P. R. China.

**Keywords:** Nasopharyngeal carcinoma, induction chemotherapy, efficacy, toxicity, nedaplatin

## Abstract

**Aims:** This study aimed to investigate the clinical value of induction chemotherapy (IC) with docetaxel, 5-fluorouracil plus nedaplatin followed by concurrent chemoradiotherapy (CCRT) with nedaplatin for locoregional advanced nasopharyngeal carcinoma (NPC).

**Materials and Methods:** In total, 269 patients diagnosed with locoregional advanced NPC between June 2012 and June 2017 were retrospectively included and divided into two groups: IC (docetaxel plus nedaplatin and 5-fluorouracil) followed by nedaplatin-based CCRT (TNF + N group, n = 146) and IC (docetaxel plus cisplatin and 5-fluorouracil) followed by cisplatin-based CCRT (TPF + P group, n = 123). The Kaplan-Meier method and Cox proportional hazards model were applied to analyse survival and prognosis. After propensity score-matched (PSM), 113 patients remained in each group. Toxicities were compared between the two groups using the Chi-square test or Fisher's exact test.

**Results:** The overall survival (OS), progression-free survival (PFS), distant metastasis-free survival (DMFS), and locoregional relapse-free survival (LRRFS) rates of the TNF + N and TPF + P groups were 90.7% *vs.* 92.3% (*P* = 0.315), 78.9% *vs.* 79.4% (*P* = 0.715), 82.4% *vs.* 85.1% (*P* = 0.441) and 96.1% *vs.* 93.3% (*P* = 0.414), respectively, with no significant difference in 3-year survival outcome between the two groups, and this outcome was confirmed after using PSM analyses. In the PSM cohort, a significant higher frequency of grade 3/4 vomiting was observed in the TPF + P group compared to the TNF + N group (22.1% *vs.* 0%, *P* = 0.000). However, 15.9% of patients in the TNF + N group had grade 3/4 thrombocytopenia in comparison with 6.2% in the TPF + P group (*P* = 0.020).

**Conclusions:** The TNF regimen followed by CCRT with nedaplatin is an alternative treatment strategy to the standard TPF regimen followed by CCRT with cisplatin for patients with locoregional advanced NPC.

## Introduction

Nasopharyngeal carcinoma (NPC) is a common head and neck cancer in southern China. The International Agency for Research of Cancer estimated there were approximately 129,000 patients worldwide in 2018, and 47.7% of those occurred in China 1, 2.

The main treatment for locoregional advanced NPC is cisplatin-based concurrent chemoradiotherapy (CCRT) due to its unique anatomy and highly radio-sensitivity 3-7. Dozens of publications show that 27%~45% of patients suffer grade 3/4 gastrointestinal acute toxicities as well as nephrotoxicity and ototoxicity in the long-term follow-up, which decreases treatment compliance and affects the outcomes of patients 8-10. Therefore, the identification of platinum with less toxicity to replace cisplatin is an urgent need and a research hot spot.

Some studies focusing on nedaplatin-based CCRT alone or carboplatin-based CCRT alone in locoregional advanced NPC showed equivalent outcomes with less toxicity and were greatly promising 9, 11. Chitapanarux Imjai and colleagues 11 made an effort to minimise the toxicities caused by cisplatin-based CCRT by replacing cisplatin with carboplatin, showing less gastrointestinal toxicities in the carboplatin arm. Inversely, subsequent research 12 revealed that the addition of carboplatin to radiotherapy did not benefit locoregional advanced NPC patients, which indicated carboplatin was not appropriate for CCRT. Mai HQ's study indicated nedaplatin-based CCRT was an alternative treatment strategy to cisplatin-based CCRT in locoregional advanced NPC 9. However, patients were treated with CCRT alone and without induction chemotherapy (IC) or adjuvant chemotherapy (AC) in Mai's study 9. Recently, several studies demonstrated IC could further improve the efficacy and benefit the survival of patients with locoregional advanced NPC 8, 13-16. IC followed by CCRT is recommended as Category 2A for locoregional advanced NPC, which is the same recommendation level as CCRT followed by AC according to NCCN guidelines.

Based on TAX 323 and TAX 324 studies 17, 18, the docetaxel plus cisplatin and 5-fluorouracil (TPF) regimen is the first line for IC in head and neck cancer. Meanwhile, the TPF regimen is considered the Category 1 recommendation for patients with Epstein Barr virus (EBV)-associated NPC since previous studies confirmed its superior efficacy 8, 16. Notably, the cisplatin-based TPF regimen is well-known for severe side effects such as haematological toxicities and gastrointestinal reactions 8. It is unknown whether a nedaplatin-based triple-drug IC regimen could achieve similar outcomes with less toxicity compared to the cisplatin-based TPF regimen. Therefore, this retrospective study aimed to explore the clinical value of IC with docetaxel, 5-fluorouracil plus nedaplatin or cisplatin followed by CCRT with nedaplatin or cisplatin in patients with locoregional advanced NPC.

## Materials and Methods

### Patient population

Patients treated at the Affiliated Cancer Hospital & Institute of Guangzhou Medical University with locoregional advanced NPC between June 2012 and June 2017 were retrospectively investigated. The clinical stage was restaged by senior doctors according to the 8^th^ edition UICC/AJCC Classification (2017). The pathological type was determined according to the WHO histological classification of NPC. The performance status was confirmed by the Eastern Cooperative Oncology Group (ECOG) standard.

The inclusion criteria were as follows: (1) pathologically diagnosed with WHO type II or III NPC, (2) clinical stage III or IVa, (3) ECOG: 0 to 1, (4) age: 18 ~ 70 years old, (5) treated with TNF (docetaxel + nedaplatin + 5-fluorouracil) followed by N (nedaplatin)-based CCRT (TNF + N group) or TPF followed by P (cisplatin)-based CCRT (TPF + P group). The exclusion criteria were as follows: (1) a history of other malignant tumours, (2) no complete clinical data, (3) received concurrent chemotherapy with platinum drugs different from those used in IC, (4) with severe heart, liver, kidney, lung and other diseases.

**Figure 1:**
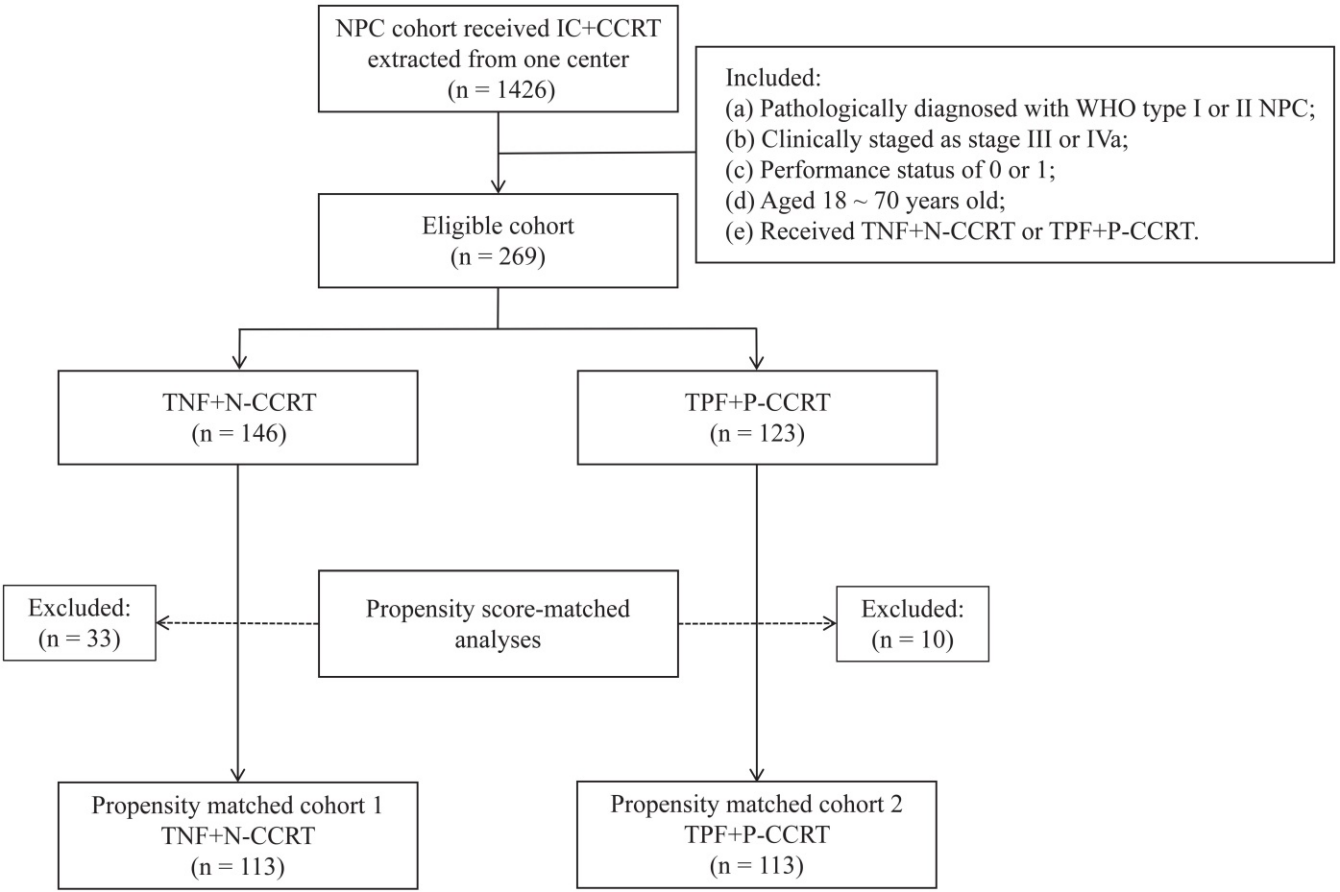
Flow chart of patient selection. NPC, nasopharyngeal carcinoma; IC, induction chemotherapy; CCRT, concurrent chemoradiotherapy; TNF, docetaxel, nedaplatin and 5-fluorouracil; TPF, docetaxel, cisplatin and 5-fluorouracil.

From June 2012 to June 2017, 1426 NPC patients were treated with IC followed by CCRT at our hospital. After screening with the inclusion and exclusion criteria, 269 patients were collected, with 146 in the TNF + N group and 123 in the TPF + P groups (**Figure 1**). This study was approved by the Research Ethics Committee of the Affiliated Cancer Hospital & Institute of Guangzhou Medical University.

### Radiotherapy

Simultaneous intensity-modulated radiotherapy (IMRT) was performed for all patients. Patients were immobilised in the supine position with a head and neck thermoplastic mask. Contrast and non-contrast CT scan were performed in 3 mm per slice with CT simulator from the vertex to 2 cm below the clavicle. Images were imported to the Pinnacle 9.10 treatment planning system (Amsterdam, Netherlands). The target volumes were delineated according to the Sun Yat-sen University Cancer Center institutional treatment protocol 19. Gross tumour volumes were determined by CT/MRI images, physical examination and nasal endoscopy results. The dose to nasopharynx gross tumour volume (GTVnx) and lymph node gross tumour volume (GTVnd) were 68 ~ 70 Gy. The dose to high-risk clinical target volume (CTV1) and low-risk clinical target volume (CTV2) were 60 ~ 62 Gy and 54 ~ 56 Gy, respectively. All patients were irradiated in 30 ~ 33 fractions, once a day for five days per week.

### Chemotherapy

For patients in the TPF + P group, 1 ~ 4 cycles of IC were delivered with docetaxel (60 ~ 75 mg/m^2^, day 1), cisplatin (60 ~ 75 mg/m^2^, day 1) and 5-fluorouracil (500 ~ 600 mg/m^2^, per day, days 1 ~ 5) every 3 weeks per cycle. Concurrent chemotherapy was administered with high-dose cisplatin (80 mg/m^2^, day 1) every 3 weeks or low-dose cisplatin (30 mg/m^2^, day 1) every week during radiotherapy.

For patients in the TNF + N group, the same drugs used for the TPF + P group were administered, except cisplatin was replaced by nedaplatin (at the same dose).

### Follow-up

After completion of treatment, patients were followed up at an interval of 3 months in the first 2 years, every 6 months in years 3 ~ 5, and every year thereafter. Follow-up visits consisted of physical examination, chest radiography, abdominal ultrasound, electronic nasopharyngoscopy and head and neck MRI. Toxicities were evaluated based on the National Cancer Institute Common Terminology Criteria for Adverse Events (NCI-CTCAE, version 4.0) 20. The follow-up time was calculated from the date of diagnosis to the day of last follow-up or death. Overall survival (OS) was defined as the interval from the date of diagnosis to death from any cause or to the last follow-up. Progression-free survival (PFS), distant metastasis-free survival (DMFS) and locoregional relapse-free survival (LRRFS) were defined as the time from the date of diagnosis to disease progression, distant metastasis, or locoregional relapse, respectively.

The acute toxic side effects and survival data were documented for all patients in outpatient and inpatient medical records systems.

### Statistical analysis

A propensity score-matched (PSM) analysis 21 was applied to balance the potential prognosis factors. At a calliper of 0.05, the PSM was generated with a one-to-one nearest neighbour matching algorithm. R (ver. 3.6.3; Auckland, New Zealand) was used to perform PSM analyses. All statistical analyses were performed using Statistical Package for the Social Sciences (ver. 25.0; SPSS, Chicago, IL, USA). The Chi-square test or Fisher's exact test was used to analyse patients' baseline characteristics and acute toxic side effects. Survival was analysed using the Kaplan-Meier method and the log-rank test. Multivariate analysis by the Cox proportional hazards model using the Forward: LR method was performed to test potentially important prognostic factors. For all statistical analyses, a *P* < 0.05 was deemed to indicate statistical significance.

## Results

### Patient characteristics

In the whole eligible cohort, 269 patients with a median age of 47 years (19 ~ 70 years) were included, with 146 patients in the TNF + N group and 123 in the TPF + P group. After PSM, 113 patients remained in each group, and baseline characteristics were well balanced between the two groups (*P* > 0.05) (**Table 1**).

### Survival analyses

#### All patients' included

**Figure 2:**
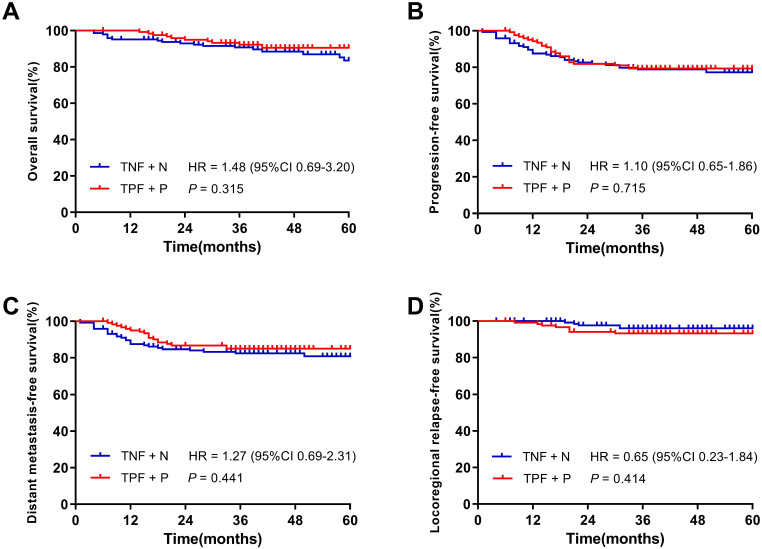
Kaplan-Meier survival curves of the TNF + N group and TPF + P group in patients before matching. **A.** Overall survival; **B.** Progression-free survival; **C.** Distant metastasis-free survival; **D.** Locoregional-free survival.

With a median follow-up of 40 months (4 ~ 94 months) in all patients, the 3-year OS, PFS, DMFS and LRRFS rates were 91.9%, 79.1%, 83.7% and 94.8%, respectively. The OS, PFS, DMFS and LRRFS rates of patients in the TNF + N and TPF + P groups were 90.7% *vs.* 92.3% (*P* = 0.315), 78.9% *vs.* 79.4% (*P* = 0.715), 82.4% *vs.* 85.1% (*P* = 0.441) and 96.1% *vs.* 93.3% (*P* = 0.414), respectively, showing no significant difference in the 3-year survival rates between the two groups (**Figure 2**).

#### Patients after propensity score-matched

**Figure 3:**
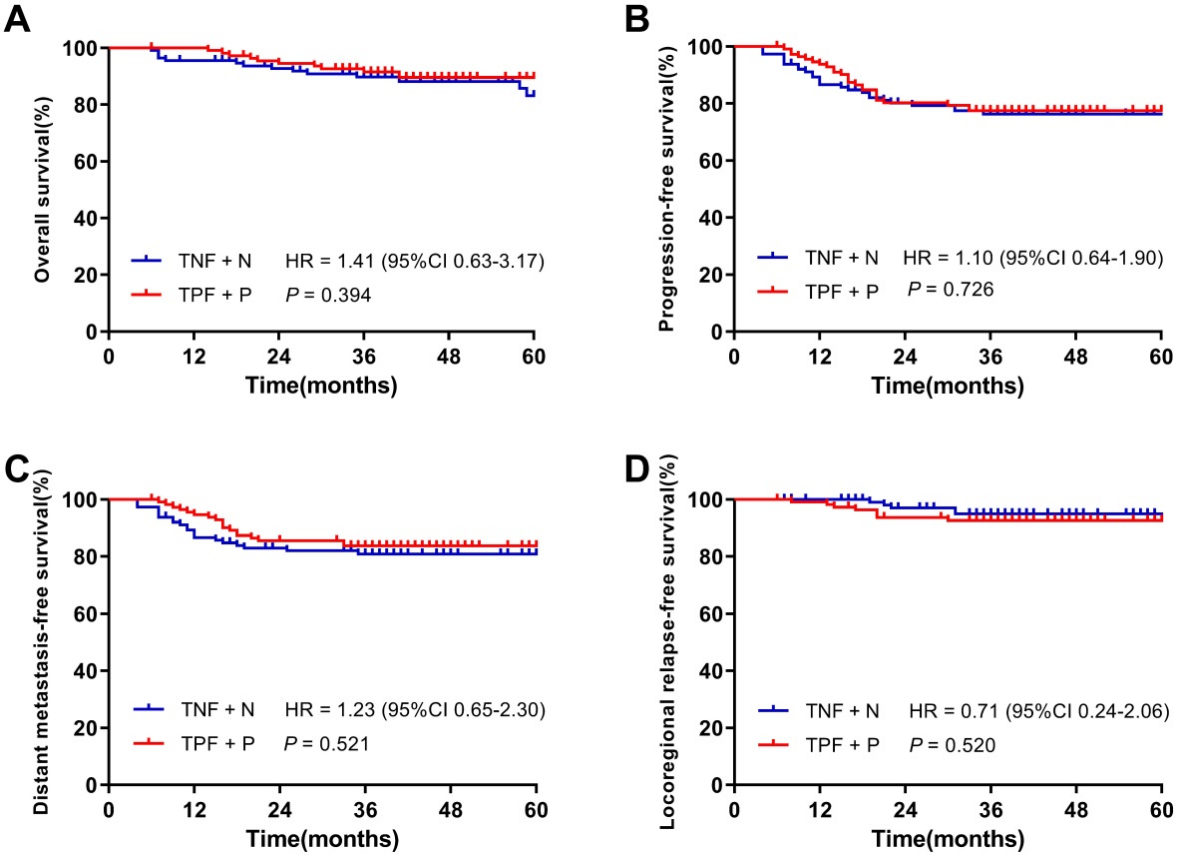
Kaplan-Meier survival curves of the TNF + N group and TPF + P group in patients after matching. **A.** Overall survival; **B.** Progression-free survival; **C.** Distant metastasis-free survival; **D.** Locoregional-free survival.

After PSM, 226 patients were identified and there were 113 patients in each cohort. With a median follow-up of 39.5 months (6 ~ 94 months) of 226 patients, the 3-year OS, PFS, DMFS and LRRFS rates were 90.6%, 76.9%, 82.3% and 93.8%, respectively. **Figure 3** demonstrated the 3-year OS, PFS, DMFS and LRRFS rates of patients in the TNF + N and TPF + P groups were 89.8% *vs.* 91.5% (*P* = 0.394), 76.3% *vs.* 77.5% (*P* = 0.726), 80.9% *vs.* 83.7% (*P* = 0.521) and 95.0% *vs.* 92.7% (*P* = 0.520), respectively, showing similar 3-year survival rates between the two groups.

#### Multivariate survival analysis

Before PSM, the variables entered into the multivariate analysis included gender, age, T stage, N stage, clinical stage, cycles of IC, cumulative doses of platinum-based CCRT, BMI, smoking status, LDH and treatment groups. Multivariate survival analysis revealed the independent prognostic factors for OS included age (*P* = 0.031), clinical stage (*P* = 0.001) and LDH (*P* = 0.007). Clinical stage and LDH were the independent prognostic factors for both PFS and DMFS (**Table 2**).

Furthermore, multivariate survival analysis was performed after PSM. The variables entered into the multivariate analysis were the same as before PSM. **Table 3** showed that age (*P* = 0.027), clinical stage (*P* = 0.001) and LDH (*P* = 0.003) were the independent prognostic factors for OS. Similarly, the independent prognostic factors for PFS and DMFS included clinical stage and LDH after PSM.

### Toxicity

Toxicities were analysed in the PSM cohort (**Table 4**). In terms of non-haematologic toxicities, grade 3/4 vomiting was more common in the TPF + P group than that in the TNF + N group (22.1% *vs.* 0.0%, *P* = 0.000). Grade 3/4 radiation-induced oral mucositis tends to be worse in the TPF + P group compared to the TNF + N group (31.9% *vs.* 21.2%, *P* = 0.071). No significant differences were found in the incidences of weight loss, hypoalbuminemia, acute radiation-induced oral mucositis or liver and kidney function impairment between the two groups (*P* > 0.05). For haematologic toxicities, it was noticed that grade 3/4 thrombocytopenia in the TNF + N group was significantly higher than that in the TPF + P group (15.9% *vs.* 6.2%, *P* = 0.020). No significant differences were found in the incidences of leukopenia, neutropenia and anaemia between the two groups (*P* > 0.05).

Further investigation of the contribution of IC or CCRT to the toxicity was performed in the two groups (**Table 5 and Table 6**). Similarly, regardless of IC or CCRT, more patients suffered from grade 3/4 vomiting in the TPF + P group than those in the TNF + N group (12.4% *vs.* 0.0%, *P* = 0.000; 21.2% *vs.* 0.0%, *P* = 0.000, respectively). Although no significant difference in the incidence of grade 3/4 thrombocytopenia during IC was found between the TPF + P and TNF + N groups (0.9% *vs.* 0.0%, *P* = 1.000), the incidence during CCRT was significantly higher in the TNF + N group than that in the TPF + P group (15.9% *vs.* 5.3%, *P* = 0.010). The incidence of grade 3/4 leucopenia and neutropenia during CCRT were higher in the TNF + N group than those in the TPF + P group (36.3% *vs.* 22.1%, *P* = 0.019; 31.0% *vs.* 16.8%, *P* = 0.013, respectively). There was no significant differences in anaemia, weight loss, hypoalbuminemia, or liver and kidney function impairment during IC or CCRT between the two groups (*P* > 0.05).

## Discussions

This retrospective study revealed first that TNF regimen followed by CCRT with nedaplatin was an alternative treatment strategy to the standard TPF regimen followed by CCRT with cisplatin for patients with locoregional advanced NPC. The TNF + N group had a similar survival outcome and much less grade 3/4 vomiting compared to the TPF + P group, while the incidence of grade 3/4 thrombocytopenia was higher in the TNF + N group.

The goals of treatment for NPC are to improve survival and reduce the treatment-induced toxicity. Generally, the selection of the treatment regimen should be based on the effectiveness of the regimen and patient compliance. In a randomised phase III trial, Mai HQ and colleagues 9 successfully showed that nedaplatin-based CCRT was non-inferior to cisplatin-based CCRT in 2-year PFS for patients with stage II-IVB nasopharyngeal carcinoma. However, a subsequent comment 22 pointed out that it was premature to draw the conclusion that nedaplatin was a suitable alternative to cisplatin combined with radiotherapy for NPC patients. In the present study, comparison was made between nedaplatin and cisplatin on the basis of the standard TPF regimen, which was different from previous studies 9, 23-25. Our study showed that 3-year OS, PFS, DMFS and LRRFS in the eligible cohort of TNF + N and TPF + P groups were 90.7% and 92.3%, 78.9% and 79.4%, 82.4% and 85.1%, 96.1% and 93.3%, respectively. These survival outcomes were consistent with a randomised phase III trial 8, in which the 3-year OS, PFS, DMFS and LRRFS in NPC patients treated with TPF IC followed by CCRT were 92%, 80%, 90% and 92%, respectively. We also found in this study that age, clinical stage, and LDH were independent prognostic factors, consistent with earlier studies 26-28.

The toxicity profiles of both treatment regimens were as expected based on previous studies of cisplatin and nedaplatin 9, 23, 24. Nedaplatin-based regimen could cause more gastrointestinal toxicities than cisplatin-based regimen. Among all patients in the PSM cohort in this study, none of patients suffered grade 3/4 vomiting in the TNF + N group compared to 22.1% in the TPF + P group, which significantly improved the compliance of patients to treatment. Hu Fujun's study 24 focusing on IC with 5-fluorouracil plus cisplatin or nedaplatin followed by CCRT in NPC demonstrated that no one in the nedaplatin group suffered from grade 3/4 vomiting, which is consistent with our study. For toxicities during CCRT, it is noticed that grade 3/4 vomiting in the TPF + P group reached 21.2%, which is also comparable to Mai HQ's work 9. However, no statistically significant difference in weight loss or hypoalbuminemia was found between the two groups regarding IC or CCRT period (*P* > 0.05). In clinical practice, nutritional support would be utilised or enhanced once NPC patients suffer from severe vomiting 29. This may be the reason why these two indexes demonstrated no statistically significant difference between the two groups. In terms of haematological toxicity, previous studies showed approximately 40% of grade 3/4 leucopenia or neutropenia occurred in NPC patients treated with a nedaplatin-based IC regimen followed by CCRT 23, 24. Similarly, the present study found that 38.9% of patients suffered from grade 3/4 leucopenia among NPC patients in the TNF + N group. Thrombocytopenia is another significant haematological toxicity caused by nedaplatin. During IC, no statistically significant difference was found in the incidence of grade 3/4 thrombocytopenia between the two groups (*P* = 1.000), but the frequency of grade 3/4 thrombocytopenia in the TNF + N group during CCRT (15.9%, 18/113) was significantly different than that in the TPF + P group (5.3%, 6/113) (*P* = 0.010). This difference was reflected during CCRT, suggesting the potential risk caused by the delayed platelet toxicity of nedaplatin.

This study has several limitations. First, it was a retrospective study with inevitable bias, although PSM was used to reduce interference factors. Second, there is a lack of results regarding quality of life and patient compliance. In view of these limitations, the relevant conclusions should be further confirmed by prospective randomised clinical studies with a large sample size.

In summary, this study demonstrated the TNF + N group has a similar survival outcome and less grade 3/4 vomiting than the TPF + P group, suggesting that the TNF regimen followed by CCRT with nedaplatin is an alternative treatment strategy to the standard TPF regimen followed by CCRT with cisplatin for patients with locoregional advanced NPC.

## Figures and Tables

**Table 1:** Patient characteristics in the TNF + N group and TPF + P group before and after matching

Characteristics	Eligible cohort before PSM [cases (%)]		*P*	PSM cohort [cases (%)]		*P*
	TNF + N	TPF + P		TNF + N	TPF + P	
**Total**	**146 (54.3)**	**123 (45.7)**		**113 (50.0)**	**113 (50.0)**	
**Age (years)**			0.033			0.594
< 47	64 (43.8)	70 (56.9)		56 (49.6)	60 (53.1)	
≥ 47	82 (56.2)	53 (43.1)		57 (50.4)	53 (46.9)	
**Gender**			0.964			0.494
Male	116 (79.5)	98 (79.7)		94 (83.2)	90 (79.6)	
Female	30 (20.5)	25 (20.3)		19 (16.8)	23 (20.4)	
**Histology**			0.223			0.307
WHO II	4 (2.7)	7 (5.7)		3 (2.7)	6 (5.3)	
WHO III	142 (97.3)	116 (94.3)		110 (97.3)	107 (94.7)	
**T stage**			0.467			0.182
T1-2	50 (34.2)	37 (30.1)		27 (23.9)	36 (31.9)	
T3-4	96 (65.8)	86 (69.9)		86 (76.1)	77 (68.1)	
**N stage**			0.750			0.872
N0-1	32 (21.9)	25 (20.3)		24 (21.2)	25 (22.1)	
N2-3	114 (78.1)	98 (79.7)		89 (78.8)	88 (77.9)	
**Clinical stage**			0.059			0.790
III	88 (60.3)	60 (48.8)		62 (54.9)	60 (53.1)	
IVa	58 (39.7)	63 (51.2)		51 (45.1)	53 (46.9)	
**IC cycle**			0.924			0.344
1-2	87 (59.6)	74 (60.2)		63 (55.8)	70 (61.9)	
3-4	59 (40.4)	49 (39.8)		50 (44.2)	43 (38.1)	
**Platinum dose of CCRT (mg/m^2^)**			0.137			0.287
< 200	71 (48.6)	71 (57.7)		55 (48.7)	63 (55.8)	
≥ 200	75 (51.4)	52 (42.3)		58 (51.3)	50 (44.2)	
**BMI**			0.946			1.000
< 18.5	11 (7.5)	9 (7.3)		8 (7.1)	8 (7.1)	
≥ 18.5	135 (92.5)	114 (92.7)		105 (92.9)	105 (92.9)	
**Smoking**			0.051			0.680
Yes	45 (30.8)	52 (42.3)		41 (36.3)	44 (38.9)	
No	101 (69.2)	71 (57.7)		72 (63.7)	69 (61.1)	
**LDH level (U/L)**			0.173			0.623
< 245	128 (87.7)	114 (92.7)		103 (91.2)	105 (92.9)	
≥ 245	18 (12.3)	9 (7.3)		10 (8.8)	8 (7.1)	

IC, induction chemotherapy; CCRT, concurrent chemoradiotherapy; BMI, body mass index; LDH, lactate dehydrogenase; PSM, propensity score-matched.

**Table 2:** Multivariate analysis of the OS, PFS and DMFS in the eligible cohort before PSM

Endpoint	Variable	Hazard ratio	95% CI	*P*
OS	Age (< 47 *vs.* ≥ 47)	2.34	(1.08-5.06)	0.031
	Clinical stage (III *vs.* IVa)	4.07	(1.72-9.61)	0.001
	LDH (< 275 *vs.* ≥ 275)	3.11	(1.37-7.06)	0.007
PFS	Clinical stage (III *vs.* IVa)	2.06	(1.21-3.53)	0.008
	LDH (< 275* vs.* ≥ 275)	3.26	(1.77-6.02)	0.000
DMFS	Clinical stage (III* vs.* IVa)	2.51	(1.32-4.79)	0.005
	LDH (< 275 *vs.* ≥ 275)	3.49	(1.77-6.86)	0.000

PSM, propensity score-matched; OS, overall survival; PFS, progression-free survival; DMFS, distant metastasis-free survival.

**Table 3:** Multivariate analysis of the OS, PFS and DMFS in the PSM cohort

Endpoint	Variable	Hazard ratio	95% CI	*P*
OS	Age (< 47 *vs.* ≥ 47)	2.56	(1.12-5.87)	0.027
	Clinical stage (III *vs.* IVa)	6.13	(2.09-17.95)	0.001
	LDH (< 275 *vs.* ≥ 275)	4.09	(1.61-10.39)	0.003
PFS	Clinical stage (III *vs.* IVa)	2.48	(1.38-4.45)	0.002
	LDH (< 275 *vs.* ≥ 275)	3.97	(2.02-7.83)	0.000
DMFS	Clinical stage (III *vs.* IVa)	3.45	(1.68-7.11)	0.001
	LDH (< 275 *vs.* ≥ 275)	4.13	(1.95-8.74)	0.000

PSM, propensity score-matched; OS, overall survival; PFS, progression-free survival; DMFS, distant metastasis-free survival; IC, induction chemotherapy; LDH: lactate dehydrogenase.

**Table 4:** Incidence of acute toxicities during treatment in patients after matching by type and grade

Acute Toxicities	TNF + N (n = 113)		TPF + P (n = 113)		*χ^2^*	*P*
	Grade 0-2, n (%)	Grade 3-4, n (%)	Grade 0-2, n (%)	Grade 3-4, n (%)		
**Haematologic**						
Leucopenia	69 (61.1)	44 (38.9)	82 (72.6)	31 (27.4)	3.373	0.066
Neutropenia	76 (67.3)	37 (32.7)	84 (74.3)	29 (25.7)	1.370	0.242
Anaemia	101 (89.4)	12 (10.6)	103 (91.2)	10 (8.8)	0.201	0.654
Thrombocytopenia	95 (84.1)	18 (15.9)	106 (93.8)	7 (6.2)	5.442	0.020
**Non-Haematologic**						
Vomiting	113 (100.0)	0 (0.0)	88 (77.9)	25 (22.1)	28.109	0.000
Weight loss	110 (97.3)	3 (2.7)	110 (97.3)	3 (2.7)	N/A	1.000
Hypoalbuminemia	112 (99.1)	1 (0.9)	113 (100.0)	0 (0.0)	N/A	1.000
Mucositis	89 (78.8)	24 (21.2)	77 (68.1)	36 (31.9)	3.267	0.071
Liver/Kidney damage	112 (99.1)	1 (0.9)	113 (100.0)	0 (0.0)	N/A	1.000

N/A, not applicable.

**Table 5:** Incidence of acute toxicities during IC in patients after matching by type and grade

Acute Toxicities	TNF + N (n = 113)		TPF + P (n = 113)		*χ^2^*	*P*
	Grade 0-2, n (%)	Grade 3-4, n (%)	Grade 0-2, n (%)	Grade 3-4, n (%)		
**Haematologic**						
Leucopenia	101 (89.4)	12 (10.6)	103 (91.2)	10 (8.8)	0.201	0.654
Neutropenia	90 (79.6)	23 (20.4)	93 (82.3)	20 (17.7)	0.258	0.611
Anaemia	110 (97.3)	3 (2.7)	112 (99.1)	1 (0.9)	N/A	1.000
Thrombocytopenia	113 (100.0)	0 (0.0)	112 (99.1)	1 (0.9)	N/A	1.000
**Non-Haematologic**						
Vomiting	113 (100.0)	0 (0.0)	99 (87.6)	14 (12.4)	14.925	0.000
Weight loss	112 (99.1)	1 (0.9)	111 (98.2)	2 (1.8)	N/A	1.000
Hypoalbuminemia	112 (99.1)	1 (0.9)	113 (100.0)	0 (0.0)	N/A	1.000
Liver/Kidney damage	112 (99.1)	1 (0.9)	113 (100.0)	0 (0.0)	N/A	1.000

IC, induction chemotherapy; N/A, not applicable.

**Table 6:** Incidence of acute toxicities during CCRT in patients after matching by type and grade

Acute Toxicities	TNF + N (n = 113)		TPF + P (n = 113)		*χ^2^*	*P*
	Grade 0-2, n (%)	Grade 3-4, n (%)	Grade 0-2, n (%)	Grade 3-4, n (%)		
**Haematologic**						
Leucopenia	72 (63.7)	41 (36.3)	88 (77.9)	25 (22.1)	5.479	0.019
Neutropenia	78 (69.0)	35 (31.0)	94 (83.2)	19 (16.8)	6.229	0.013
Anaemia	102 (90.3)	11 (9.7)	100 (88.5)	13 (11.5)	0.186	0.666
Thrombocytopenia	95 (84.1)	18 (15.9)	107 (94.7)	6 (5.3)	6.713	0.010
**Non-Haematologic**						
Vomiting	113 (100.0)	0 (0.0)	89 (78.8)	24 (21.2)	26.851	0.000
Weight loss	110 (97.3)	3 (2.7)	110 (97.3)	3 (2.7)	N/A	1.000
Hypoalbuminemia	112 (99.1)	1 (0.9)	113 (100.0)	0 (0.0)	N/A	1.000
Mucositis	89 (78.8)	24 (21.2)	77 (68.1)	36 (31.9)	3.267	0.071
Liver/Kidney damage	112 (99.1)	1 (0.9)	113 (100.0)	0 (0.0)	N/A	1.000

CCRT, concurrent chemoradiotherapy; N/A, not applicable.
